# Evaluating effects of IBEM-U on decreasing alcohol consumption and heavy episodic drinking among university students in Colombia: Protocol for a randomized control trial

**DOI:** 10.1016/j.conctc.2023.101075

**Published:** 2023-01-23

**Authors:** Juliana Mejía-Trujillo, Augusto Pérez-Gómez, Hein de Vries, Liesbeth Mercken

**Affiliations:** a Department of Health Promotion, Maastricht University, the Netherlands; b Corporación Nuevos Rumbos, Bogotá, Colombia; c Department of Health Psychology, Open University, Heerlen, the Netherlands

**Keywords:** University students, Prevention, Effects, Heavy episodic drinking, Evaluation

## Abstract

**Background:**

Alcohol misuse is a serious problem among university students in Colombia as well as in other Latin American countries. Studies show consistently that this population presents the highest rates of alcohol use. Despite such a situation, there is a lack of preventive programs for university students in this region of the world. The purpose of this paper is to present the protocol to evaluate a preventive strategy called IBEM-U, based on Motivational Interviewing and the I-Change Model.

**Method:**

This protocol shows how the evaluation of the effectiveness of the IBEM-U program will be carried out. A randomized control trial with a within-subjects design with one follow-up at six months after the post-test will be implemented. The comparison group will receive an alternative program similar in length but focusing on another issue. Around 1000 participants over 18 years of age, from at least six different universities around the country, will be recruited.

**Results:**

It is expected that the program will be effective in reducing past month alcohol consumption up to 15% in the experimental group as the main outcome. Secondary and tertiary outcomes include decreasing heavy episodic drinking and increasing knowledge, awareness, risk perception, attitude, self-efficacy, intention, and action planning, regarding heavy episodic drinking.

**Conclusion:**

IBEM-U can be considered a highly appropriate approach for reducing alcohol misuse among university students. The main reasons for these results are the self-imposed goals based on long-term purposes, that could be seriously affected by the ingestion of high amounts of alcohol.

## 1 Introduction

### 1.1 The problem of alcohol consumption

Alcohol misuse leads to several mental, physical, academic, and social problems in young adults. Among those problems, the most relevant are depression and suicidal ideation [1]; negative impacts on the immune and digestive systems, damage of the liver resulting in fibrosis or cirrhosis, as well as the link to 25 different types of cancer in the long term [[2], [3], [4]]; higher risk of unwanted pregnancies and sexual transmission infections [5,6]; a progressive decline in cognitive skills and processes of memory and planning, as well as a loss of capacity for decision-making [8,9]; National Institute on Alcohol Abuse and Alcoholism [ [10,11]. Regarding social problems, prior studies have found that high levels of drinking, especially among youth, can act as a risk factor for becoming a victim or perpetrator of violence (Pan American Health Organization [12].

Although Heavy Episodic Drinking^1^ (HED – defined as ten or more standard drinks per occasion at least once in the past month) has decreased worldwide (diminishing from 47.6% in 2000 to 43% in 2016 for current drinkers, and from 22.6% in 2000 to 18.2% in 2016 for heavy episodic drinkers), alcohol use among young adults remains a public health problem (World Health Organization [13]. The Americas region (North and Latin America) has the second highest rates of alcohol use among young adults aged between 20 and 24 years (38.2%), after the European region (43.8%) [14]. Data on excessive drinking are particularly high among university students in the Americas, pointing out that HED in the past month, corresponded to 18.6% among Latin American university students; Colombia, Uruguay, Ecuador, and Brazil have the highest levels of alcohol consumption (Organization of American States [15]. The same report states that between 60% and 87% of the Latin American university population perceived alcohol use as a severe risk to for intoxication; nevertheless, they continue excessive drinking. In Colombia, university students have the highest rates of alcohol use: 55.4% report using alcohol in the past month, compared with 38.4% of 18 to 24-year-olds and 39.7% of 25 to 34-year-olds in the general population (Comunidad Andina de Naciones [16].

HED is a common practice among university students in Colombia; the usual pattern according to Ref. [17] is biweekly or monthly (58.1% of those who show HED). Among students, 85% drink especially in discos and bars, mainly with friends, and about 30% drink at friends’ homes. There are no significant differences in drinking patterns between young men and women [17,18]. The involvement of the students in HED has several reasons, such as widespread availability of alcohol nearby the facilities, high social pressure to drink, leisure time between classes, and increased stress due to new academic demands [10,19]. In spite of the difficulty to find effective strategies to work with this population, prevention appears to be the most logic and potentially most productive way to tackle the situation.

### 1.2 Preventive interventions

Alcohol use prevention, including HED, in universities has been addressed through multiple modalities. Online interventions are one such modality, although specific interventions have varied in length and delivery format [20]. Some online brief alcohol interventions have been created to deliver feedback once the person fills out a questionnaire [21]. Other interventions promote responsible decision-making regarding alcohol use: different scenarios are presented to the participant for them to choose the best option and they then receive feedback based on their choice [22]. There are also interventions which are carried out entirely by email [23]. Although those online programs reach more people compared to face-to-face interventions, they lack feedback sufficiently tailored to the participants’ realities, adjusted to what participants know and believe about alcohol and drug consumption [20]. They also have been criticized for lacking persuasive theoretical approaches, including awareness of risks about certain behaviors, especially culturally-normative behaviors such as alcohol consumption.

In this protocol we describe a new preventive intervention for university population which uses the I-Change Model [24] as theoretical framework, and Motivational Interviewing [25]; 2013 [26]; as the method. Both approaches have shown effectiveness in facilitating behavior change: In a meta-analytic review [27], found that face-to-face interventions based on Motivational Interviewing reported more extended periods of fewer problems related to alcohol abuse (up to six months); they also mention that this approach could be considered one of the most efficacious for university students who have had episodes of heavy drinking. Another meta-analysis (Kohler & Hofmann, 2014) observed that Motivational Interviewing was more effective than other approaches in reducing frequency and quantity of alcohol consumption among young people. Regarding the I-Change Model, a randomized control trial showed that those adults who received the intervention with this theoretical framework decreased 3.9 drinks weekly, compared to the control group, in a follow-up six months after the intervention (Shulz et al., 2013). Other studies have demonstrated the efficacy of this approach among other populations, such as adolescents [28], and pregnant women six months after baseline [29]. Both approaches also acknowledge the importance of raising awareness of the problems associated with alcohol misuse, increasing motivation to adopt a healthier behavior, setting goals for change, and developing action plans for enacting the desired healthy behavior. The I-Change model offers clear steps to increase awareness (e.g. to increase cognizance about the level of drinking and knowledge about the risks of drinking), to enhance motivation (e.g. by developing a positive attitude, positive norms and self-efficacy), and to develop clear action and coping plans [28,30]. Motivational interviewing will be used to provide the didactic framework for the intervention by focusing on providing empathy, developing discrepancy, rolling with resistance, and supporting self-efficacy. Both models will serve as the theoretical underpinning for this intervention IBEM-U (acronym in Spanish of *Intervención Breve basada en Entrevista Motivacional* - Brief Intervention based on Motivational Interviewing for University students).

In Colombia, the combination of these approaches with this population in an online intervention has not been used before. Moreover, there are only six evaluated programs, all of which are addressed to adolescents from secondary school; programs for university students do not exist in the country [31,32].

IBEM -U is an online prevention program for university students and is based on our previously created and evaluated face-to-face IBEM program for adolescents; IBEM showed significant effects on decreasing alcohol consumption [33,34]. A pilot was conducted in 2020 with 60 students, and adjustments were made mainly to the platform. This paper describes the study protocol of the IBEM-U program (main intervention) to assess its effects on decreasing alcohol consumption and heavy episodic drinking among university students.

## 2 Methodology

### 2.1 Study design

The effectiveness of IBEM-U to reduce problem alcohol use will be analyzed using a randomized control trial with a within-subjects design with one follow up at six months after the post-test. The intervention group will receive IBEM-U, the comparison group will receive an alternative program similar in length but focusing on learning strategies (EDA – acronym in Spanish of *Estrategias De Aprendizaje* –), aimed at promoting the use of strategies to facilitate the learning process in general. A baseline measurement (T0) will be obtained before randomization; a second measurement will occur at the end of the program (T1) – (three months after baseline); and a follow-up at six months after the second measurement (T2) (nine months after baseline).

### 2.2 Goals and hypotheses

The main goal of the RCT is to evaluate the effects of an adaptive program aimed at reducing frequency and quantity of alcohol use, among young adults from different universities in Colombia, compared to a control group, immediately after its implementation and six months later. The primary outcome is the number of standard drinks consumed in the past month. We hypothesize that the IBEM-U program will be effective in reducing past month alcohol consumption in undergraduate and graduate adult students, up to 15% in the experimental group. Secondary outcomes are decreasing heavy episodic drinking up to 10%. Tertiary outcomes include changes related to knowledge, awareness, risk perception, attitude, self-efficacy, intention, and action planning, regarding heavy episodic drinking.

### 2.3 Participants and recruitment

For the RCT, undergraduate or graduate students will be recruited from participant universities. Inclusion criteria include being an adult over 18 years of age, agreeing to be randomly assigned to either intervention or comparison group and being able to read Spanish. Year of school, department/university, socioeconomic status, and gender will be measured as control variables. For recruitment, 103 Colombian universities have been invited to participate, through presentations and emails. Some universities will need a formal agreement outlining the particulars of the intervention and how assessments among participants will be done; others will extend the invitation to students for them to participate by themselves. Multiple strategies will be used to recruit participants; they will include a call for participation sent by the universities, through social media, institutional emails, and promotional videos. Invitations will include the link for registration, where we will ask for name, email, and phone number. The information will be hosted in the [Corporación Nuevos Rumbos] server with only the research team having access. The researchers will contact the registered participants and give detailed information about the procedure and send informed consent forms for participation, inviting them to accept or refuse the intervention. The consent form specifies that the collected information will be confidential, that it will be used for the intervention and research goals, and that they will be randomly assigned to either IBEM-U or EDA. Individuals participating in other alcohol prevention program will be excluded. As the intervention is online, contamination is considered unlikely. Those students who agree to participate will receive a link to fill out the measurement instruments and schedule an appointment for the first meeting on one of the two programs.

### 2.4 Intervention trial

#### 2.4.1 IBEM-U

The program is based on motivational principles as used in Motivational Interviewing [25]; 2013; [26], and the I-Change Model [30].

IBEM-U is an adaptation for university students of the version for secondary adolescents comprised of two 20-min sessions that are three months apart. The process is highly interactive, in which the student and facilitator are continuously exchanging information, questions and suggestions. Every meeting requires participants to fill out two questionnaires before they start the session. These instruments will measure alcohol use and level of risk due to their pattern of consumption, as well as the I-Change Model components (for more detail, see the measures section). During the intervention, a trained facilitator will guide the student throughout the six steps described in Table 1, Table 2. The same will happen in the second meeting, but the goal and topics of conversation are different. The reader can find the step-by-step guide of the intervention with the related tools and examples in the complete manual at [www.nuevosrumbos.org].

**Table 1:** Steps to be followed during the first session of the program.

STEPS	
1. *introduction and orientation to the program:* The facilitator introduces him/herself, discusses the goal of the intervention, and confirms the informed consent and the sociodemographic data.	4. Importance and confidence scales*:* The facilitator asks the participant to rank how important it is to them to maintain low-risk alcohol assumption on a scale from 1 to 10. The facilitator discusses the participant's answer to determine the main motivations for making a change. Then he or she asks the participant to also rank their confidence to make a change or maintain a low-risk consumption from 1 to 10.
2. *Risk level:* The facilitator revises the risk level delivered by the system, based on the answers related to alcohol use prevalence and to the AUDIT instrument (WHO, 2000). The options of risk level are Low, Medium, High, or Severe, and it is displayed on the screen of the participant in form of pyramid. The facilitator explains why the participant is placed in this level and continues with the interview.	5. *Self-imposed goal and strategies:* The participant is guided to propose an action plan composed of a goal regarding alcohol use and several strategies to achieve it. The user is responsible of determining what, how and when he/she will do it.
3. *Evocation and motivators:* Answers to the AUDIT and other questions related to consumption patterns from the interview are used to discuss alcohol use in more depth. Attitudes, practices, experiences, and other perspectives about alcohol use are identified. Any myths about consumption that arise will be clarified. All this information is stored in an electronic platform. Then the facilitator asks about other activities such as free time and short- and medium-term plans. Using this information, the facilitator focuses the intervention on salient physical or psychosocial risks. Facilitator's feedback is personalized based on the conversation between the facilitator and the participant.	6. Summary: the last step of the intervention is discussing with three final questions with the participant: 1. What are the obstacles to achieving the goal? 2. What strategies can be used to cope with difficulties that arise? 3. What are the advantages of maintaining low-risk consumption?

**Table 2:** Steps to be followed during the second session of the program.

STEPS	
1. *introduction and orientation to the program:* The facilitator introduces him/herself, discusses the goal of the intervention, and confirms the informed consent and the sociodemographic data. The goal of this meeting is different to the first one, it is to do a balance of the process.	4. I*mportance and confidence scale:* The professional asks: in a 1 to 10 points scales, where 1 is “not at all” and 10 is “very important”, how important is for you to maintain a low-risk alcohol consumption? The facilitator inquiries about the choice to determine what are the main aspects to make a change. Then, he asks the same but with a scale of confidence to make a change or maintain a low-risk consumption.
2. *Summary and Risk level:* The facilitator summarizes the past session and determines the risk level to be used for the session based on the answers related to alcohol use prevalence and to the AUDIT instrument (WHO, 2000). He/she communicates the risk level and informs the reasons why the participant is placed in this level of the intervention to continue with the interview.	5. *Monitoring the process:* The participant is asked about changing or maintaining the same action plan based on his/her new answers and the barriers and facilitators identified during both sessions.
3. *Goal achievement:* Every participant is asked about the achievement of the goal he or she created at the end of the previous session. Barriers and facilitators to achieving the goal will be discussed.	6. *Summary*: A synthesis of the process is presented to the participant; the goal of this step is to increase self-efficacy. Not only verbal but non-verbal communication is important for them to fulfill their goals.

#### 2.4.2 EDA (Learning Strategies) control group

In order to ensure that all participants are able to potentially benefit from the study, the research team decided to deliver another intervention to those participating in the control group. This intervention will address another topic that is relevant to university students – study habits and how to improve them – but will not address alcohol and other drug use in order to not interfere with the study results.

This program is aimed at promoting the use of strategies to ease the apprehension, storage, understanding, and usability of information [35]. It is highly linked to the person's self-knowledge (strengths, weaknesses, motivation, etc); to the assigned task; and to her/his own learning process, which generally implies a high level of cognizance about the learning process [36]. EDA is delivered online in a one-on-one format. The first session includes a short introduction to the model and the administration of a five-items questionnaire to identify the dominant learning style of each participant (visual, aural, kinesthetic). During the intervention the facilitator shares a link with several activities they can work on until the next session.

#### 2.4.3 Professional facilitators

The facilitators of both programs (psychologists and social workers) will have received training on each strategy. IBEM-U requires 32 h of training to be implemented. Training includes information on the risks and effects of alcohol use, the steps of the intervention, and the theoretical foundations of the intervention. Role-playing is a key component of the training. A train-the-trainer model is used to train new facilitators. The trainers for this intervention will be the developers of the original program. In the past eight years they have trained more than 150 people in Colombia, Mexico, and Brazil.

### 2.5 Data analytic plan

#### 2.5.1 Sample size

Sample size was determined using a simulation-based power analysis (1000 simulations), with Superpower package [37] for R, version 4.0.3 [38]. We hypothesized means for intervention and control groups for each measure with a SD of 5 and a r = 0.2 for repeated measure variables. This will mean an estimate of an effect size for group (*f* = 0.037), measurement (*f* = 0.048) and interaction between group and measurement 1-β = 0.90. The recommended *N* for each group is 191 (see). Considering the possible attrition rate of about 50%, it is planned to recruit 764 participants.

#### 2.5.2 Random assignment

Randomization will take place at the level of the individual. Each participant will have a non-zero probability of being part of either the intervention or the comparison group. After filling out the baseline questionnaire, the platform generates a random number: odd numbers will correspond to the intervention group, even numbers will correspond to the control group. Research staff will inform the participant to which group they have been randomly assigned.

#### 2.5.3 Measures

*Sociodemographic information* will be gathered using 12 items including contact information such as name, phone number, email, as well as age, sex, socio-economic status, hometown, living town, university school, and semester (level).

*Prevalence of alcohol use*: we will ask for lifetime, past year, past month, and past week prevalence in terms of quantity and frequency (how many standard drinks [SD] do they have and how often do they consume alcohol in those time periods).

*Binge Drinking* (BD) binge drinking will be measured by asking to male participants the number of occasions on which they have had 5 or more SD and female participants the number of occasions on which they have had 4 or more SD in the past 30 days.

*Heavy episodic drinking* (HED) will be measured by asking participants how many times they have had 10 or more SD on one occasion in the last 30 days.

*Questionnaire based on I-Change Model*: Determinants of HED will be measured via 56 items about knowledge, cognizance, risk perception, attitudes, social influence, self-efficacy, intention, and action planning. These items were adapted from the Alerta Alcohol Program from the Universidad de Sevilla and Maastricht University [39,40], and [41]. Measures for each construct are as follows:

*Knowledge* is measured by 5 items (1 = true; 0 = false) to assess knowledge about HED, including questions such as whether drinking ten or more standard drinks affects the liver, increases unwanted pregnancies, or produces neurological damage.

*Cognizance* is measured by two questions on a five-item scale (1 = completely disagree, 5 = completely agree): “I'm aware that often drink 10 or more SD per occasion,” and “I'm aware that drinking 10 or more SD per occasion affects my health.”

*Risk perception* is measured by six questions about perception of danger and seriousness on a 5-point scale (1 = completely disagree; 5 = completely agree). Examples include “Having a bad academic performance is very serious; ” “The likelihood of me having a bad academic performance are high; ” “Health problems are very serious; ”and “The likelihood of me having health problems are high.”

*Attitudes* are measured by 16 questions on a 5 -point scale (1 = completely disagree; 5 = completely agree) and assess perceived rational and emotional advantages and disadvantages of HED. Examples are: “If I drink 10 or more SD I have fun with my friends”, “If I drink 10 or more SD I fit in with other people”, “If I drink 10 or more SD I have conflicts with other people”, and “If I drink 10 or more SD I'm more vulnerable”.

*Social influence* is measured by 12 items assessing social norms, modeling, and peer pressure towards HED (1 = completely disagree; 5 = completely disagree for social norms; and 1 = Never; 5 = Always, for modeling and peer pressure). Some examples include “My loved one would approve of me drinking 10 or more SD per occasion”, “How often my loved one drinks 10 or more SD per occasion?”, and “How often I feel pressured by my loved one to drink 10 or more SD per occasion?”.

*Self-efficacy* is assessed by six items about how difficult it is (1 = very difficult; 5 = very easy) not to drink 10 or more SD per occasion in different situations such as when alcohol is offered by friends, when the person is emotionally down, or when he/she is in a family meeting.

*Intention* is measured by three items to assess the intention to drink 10 or more SD in general, the next weekend and the next month (1 = “definitely not; 5 = definitely yes).

*Action planning* is measured by eight items (1 = definitely not; 5 is definitely yes) assessing plans to not have 10 or more SD in several situations and coping plans, such as drinking drinks with low alcohol content or looking for other alternatives to be relaxed when the person feels stressed.

#### 2.5.4 Data analyses

Descriptive statistics related to sociodemographic variables, alcohol use frequency and quantity, and changes in HED will be calculated. The independent variable is the participation in the IBEM-U program; dependent variables are the outcomes of the intervention in terms of alcohol use patterns and identification of factors associated with consumption (predictors = sociodemographic variables).

To determine the effect of the intervention (pre-post), we will use mixed linear models (MLM), with pre-post score, group, pre-post score*group and sociodemographics (affiliation, age, sex, program and level) as fixed effects. Participant will act as random effect, with random intercepts and random slopes for the interaction between pre-post score and group.

## 3 Discussion

University students have the highest rates of alcohol use among the general population, which leads to social, academic, and health-related problems (CAN, 2017). In Colombia there are currently no evidence-based programs for this population, and the COVID-19 pandemic brought a new challenge to the lack of initiatives for this target: the need of an effective online intervention. Prior evaluated programs targeted to university students are few in number and have not demonstrated good effect sizes; they lack feedback related to participants’ realities on alcohol and drug consumption [20]. They also lack persuasive theoretical approaches, including awareness of risks about certain behaviors, especially those that are as culturally-embedded as alcohol consumption.

The IBEM-U program is based on two motivational approaches (Motivational Interviewing and the I-Change Model) [24,25], aimed at raising awareness of the problem of alcohol misuse, increasing motivation to adopt healthy behaviors, and developing action plans to achieve the self-selected goals. The program also uses information and communication technologies (ICT) to better tailor feedback to the individual participant in order to improve the effectiveness of the intervention. The intervention will be delivered virtually by a well-trained facilitator. In Colombia, this is the first time a program with these approaches aimed at university population will be implemented.

Since the consequences of alcohol abuse on physical and mental health, as well as on academic performance, are prevalent in this population, it is imperative to develop and implement accessible programs, with theoretical foundations matching their needs, respectful of their beliefs, and effective in behavior change. The evaluation of this program will contribute to improve the health of the university students and to implement only evidence-based programs and practices in the country and in Latin America. As with previous prevention programs developed by the [Corporación Nuevos Rumbos], the evaluated program will be offered free of charge to the Colombian government; the current policy, formulated in 2019, highlights the importance to develop prevention programs for the highest-risk populations. So far there is no prevention programs in Colombia for university students.

This research has some possible limitations. First, since it is a modular intervention (using universal and/or selective prevention strategies) that is adapted to the risk level of the individual participant, many of whom will have low levels of alcohol use, it may not be possible to see significant reductions in Heavy Episodic Drinking. However, a decrease in overall alcohol consumption will still be able to be examined. Second, online interventions at universities are not common in Colombia, and it might result in high dropout rates. To counter this, there will be a raffle of several latest-technology tablets for those who complete all three measurements, and participants will receive reminders about the sessions. Third, the COVID 19 pandemic had many social impacts, not only in terms of alcohol use patterns and circumstances, but also affected recruitment for studies such as this one. The research team will try new strategies to recruit and retain participant across all three waves of data collection.

Conclusion: IBEM-U can be considered a highly appropriate approach for reducing alcohol misuse among university students. The main reason for this result is the self-imposed goals formulated based on long-term purposes that could be seriously affected by the intake of high amounts of alcohol.

### 3.1 Ethical considerations and trial registration

The project was approved by the Ethics Committee of [the institution], protocol number 050–06052021, on May 13th, 2021. All students must agree to participate in the intervention after reading the informed consent form. Data collection will be done via a password-protected electronic platform designed specifically for this project. Only members of the research team will have access to the platform. The protocol for storing data is in accordance with the [institution's] data protection policy, available at [organization Website]. The trial was registered at www.osf.if (Open Science Framework) URL: https://archive.org/details/osf-registrations-prns4-v1.

## Author contributions

JMT, APG, HdV, and LM made the conceptualization of the protocol. JMT prepared the first draft, and APG, HdV and LB reviewed, edited and wrote several versions of the manuscript. All authors read and agreed the final version.

## Declaration of competing interest

The authors report no conflict of interest.

## Footnotes

1 Based on [32] definition and taking into account the lack of agreement on a consensual definition, in this research we will define Heavy Episodic Drinking as ten or more standard drinks, at least on one occasion, in the past 30 days.

## References

[1] Foster K.T., Hicks B.M., Iacono W.G., McGue M. (2014). Alcohol use disorder in women: risks and consequences of an adolescent onset and persistent course. Psychol. Addict. Behav..

[2] Bagnardi V., Rota M., Botteri E., Tramacere I., Islami F., Fedirko V., La Vecchia C. (2015). Alcohol consumption and site-specific cancer risk: a comprehensive dose–response meta-analysis. Br. J. Cancer.

[3] Di Gennaro F., Pizzol D., Cebola B., Stubbs B., Monno L., Saracino A. (2017). Social determinants of therapy failure and multi drug resistance among people with tuberculosis: a review. Tuberculosis.

[4] Imtiaz S., Shield K.D., Roerecke M., Samokhvalov A.V., Lönnroth K., Rehm J. (2017). Alcohol consumption as a risk factor for tuberculosis: meta-analyses and burden of disease. Eur. Respir. J..

[5] Chakrapani V., Lakshmi P., Tsai A., Vijin P., Kumar P., Srinivas V. (2019). The syndemic of violence victimisation, drug use, frequent alcohol use, and HIV transmission risk behaviour among men who have sex with men: cross-sectional, population-based study in India. SSM-Population Health.

[6] Deese J., Pradhan S., Goetz H., Morrison C. (2018). Contraceptive use and the risk of sexually transmitted infection: systematic review and current perspectives. Open Access J. Contracept..

[8] Brown S.A., Tapert S.F. (2008). Adolescence and the trajectory of alcohol use: basic to clinical studies. Ann. N. Y. Acad. Sci..

[9] Lees B., Mewton L., Stapinski L.A., Squeglia L.M., Rae C.D., Teesson M. (2019). Neurobiological and cognitive profile of young binge drinkers: a systematic review and meta-analysis. Neuropsychol. Rev..

[10] National Institute on Alcohol Abuse and Alcoholism (2021). Drinking levels defined. https://www.niaaa.nih.gov/alcohol-health/overview-alcohol-consumption/moderate-inge-drinking.

[11] Pfefferbaum A., Kwon D., Brumback T., Thompson W.K., Cummins K., Tapert S.F., Brown S.A., Colrain I.M., Baker F.C., Prouty D. (2018). Altered brain developmental trajectories in adolescents after initiating drinking. Am. J. Psychiatr..

[12] Pan American Health Organization (2020). Juvenile violence in the Americas: innovative studies in research, diagnosis and prevention. https://www.paho.org/hq/dmdocuments/2010/La-violencia-juvenil-Americas-Estudios-innovadores-invest-diagn-preven.pdf.

[13] World Health Organization (2018). Alcohol: key facts. https://www.who.int/news-room/fact-sheets/detail/alcohol.

[14] Pan American Health Organization (2018). Informe sobre la situación mundial del alcohol y la salud 2018. https://iris.paho.org/bitstream/handle/10665.2/51352/OPSNMH19012_spa.pdf?sequence=1&isAllowed=y.

[15] Organization of American States (2019). Informe del Uso de Drogas en las Américas. http://cicad.oas.org/Main/ssMain/HTML%20REPORT%20DRUG%202019/mobile/index.html.

[16] Comunidad Andina de Naciones, Oficina de las Naciones Unidas contra la Droga y el Delito & Ministerio de Justicia (2017). III Estudio epidemiológico andino sobre consumo de drogas en la población universitaria, Informe Regional, 2016. http://www.odc.gov.co/PUBLICACIONES/ArtMID/4214/ArticleID/5696/III-Estudio-epidemiol243gico-andino-sobre-consumo-de-drogas-en-la-poblaci243n-universitaria-Informe-Regional-2016.

[17] Zambrano S.B., Bastidas L.E.T., Paz E.G.C. (2017). Consumo de alcohol en estudiantes universitarios colombianos. Universidad y Salud.

[18] Bastías E.M., Stiepovich J. (2014). Una revisión de los estilos de vida de estudiantes universitarios iberoamericanos. Ciencia y Enfermería.

[19] University of Nevada (2021). https://onlinedegrees.unr.edu/blog/binge-drinking-in-college/.

[20] Hennessy E.A., Tanner-Smith E.E., Mavridis D., Grant S.P. (2019). Comparative effectiveness of Brief Alcohol Interventions for college students: results from a network meta-analysis. Prev. Sci..

[21] Doumas D.M., McKinley L.L., Book P. (2009). Evaluation of two web-based alcohol interventions for mandated college students. J. Subst. Abuse Treat..

[22] The Foundation for advancing alcohol responsibility (2020). Who we are. https://www.responsibility.org/who-we-are/.

[23] Hallet J., Maycock B., Kypri K., Howat P., McManus A. (2009). Development of a web-based alcohol intervention for university studentes: processes and challenges. Drug Alcohol Rev..

[24] De Vries H., Mudde A., Leijs I., Charlton A., Vartiainen E., Buijs G., Pais Clemente M., Storm H., Gonzalez Navarro A., Nebot M., Prins T., Kremers S. (2003). The European smoking prevention framework approach (EFSA): an example of integral prevention. Health Educ. Res..

[25] Miller W., y Rollnick S. (2002).

[26] Rogers C.R. (1959).

[27] Carey K.B., Scott-Sheldon L.A., Carey M.P., DeMartini K.S. (2007). Individual level interventions to reduce college student drinking: a meta-analytic review. Addict. Behav..

[28] Martínez Montilla J.M. (2020).

[29] van der Wulp N., Hoving C., Eijmael K., Candel M., van Dalen W., de Vries H. (2014). Reducing alcohol use during pregnancy via health counseling by midwives and internet-based computer-tailored feedback: a cluster randomized trial. J. Med. Internet Res..

[30] De Vries H. (2017). An integrated approach for understanding health behavior; the I-change model as an example. Psychology and Behavioral Science International Journal.

[31] Corporación Nuevos Rumbos (2016). Guía de programas preventivos (PPP): menú de programas preventivos del consumo de sustancias psicoactivas en Colombia. https://nuevosrumbos.org/news?secnew=Publicaciones&catnew=Avispa%20Boletines.

[32] Government of Colombia (2016). Lineamientos para operar programas preventivos. Ministerio de Salud y Protección Social & UNODC.

[33] Reyes-Rodríguez M., Mejía-Trujillo J., Pérez-Gómez A., Cardozo F., Pinto C. (2018). Effectiveness of a brief intervention based on motivational interviewing in Colombian adolescents. Psicol. Teor. Pesqui..

[34] Reyes-Rodríguez M.F., Pinto-Gómez J.C., Cardozo-Macías F., Pérez-Gómez A., Mejía-Trujillo J., Toro-Bermúdez J. (2020). Evaluation of the prevention program “brief intervention based on motivational interviewing” in Colombian adolescents. Int. J. Ment. Health Addiction.

[35] Román J., y Gallego S. (1994).

[36] De la Fuente J., y Justicia F. (2003). Escala de estrategias de aprendizaje ACRA-Abreviada para alumnos universitarios. Electron. J. Res. Educ. Psychol..

[37] Lakens D., Caldwell A. (2021). Simulation-based power analysis for factorial analysis of variance designs. Advances in Methods and Practices in Psychological Science.

[38] R Core Team (2020). https://www.R-project.org/.

[39] Jander A., Crutzen R., Mercken L., de Vries H. (2014). A Web-based computer tailored game to reduce binge drinking among 16 to 18 year old Dutch adolescents: development and study protocol. BMC Publ. Health.

[40] Lima-Serrano M., Vargas-Martínez A.M., Gil-García E., Martínez-Montilla J.M., Lima-Rodríguez J.S., de Vries H. (2017). Adaptación y validación al español de cinco escalas para avaluar los determinantes del consumo de alcohol en adolescentes. An. del Sist. Sanit. Navar..

[41] Chen Y. (2018). The roles of prevention messages, risk perception, and benefit perception in predicting binge drinking among college students. Health Commun..

